# Indents

**Published:** 1902-02-15

**Authors:** 


### INDENTS.

☞ **Brief Articles of Technical or Scientific Value will receive notice and comment. Contributions invited.**

To Stop Decay at Gum Margins.—Dry the tooth carefully and apply 20 percent pyrozone for three minutes to disinfect. Then apply a full strength solution of formalin for five minutes, being careful to protect the gum from each of these chemicals. Then dry thoroughly and melt parafin and salol into the softened structure.—*Item of Interest.*

To Clarify Wax.—Melt in hot water bath, then remove from water bath and bring to a slow boil on the stove. Into the boiling wax pour a fresh egg, and stir for three or four minutes till the egg is thoroughly cooked. Strain through a cheese cloth, to remove all pieces of egg, and you will have your wax as clean and pure as when bought from the dental depot.—*Items of Interest.*

Boro-Chloretone as a Surgical Dressing.—I recently used boro-chloretone in dressing a stump after an amputation of a finger. The wound had become infected and was very painful. The pain disappeared in short order, and the patient secured the first good night's rest he had had for some time. The granulations are now healthy, and recovery is only a matter of time. As a dusting powder in minor surgical work, I find boro-chloretone very satisfactory.—Dr. E. R. Barton.

Calendula; its Properties.—Calendula stimulates proliferation of white blood corpuscles; increases the quantity of fibrine and aids its transformation into connective tissue; induces healing by first intention; promotes granulation and prevents disfiguring scars; promotes favorable cicatrization with least possible amount of suppuration; prevents or arrests gangrene and aids in healing or reproducing bone. Hence its value in the treatment of putrescent pulp canals, abscesses, pyorrhea, after extractions, etc.—W. I. Wallace, *Items of interest.*

Danger of Severing the Bicuspid Branches of the Middle Superior Dental Nerves by Opening Into the Antrum at the Canine Fossa.—J. H. Bradley, in January *Cosmos*, relates a case where he thinks the pulps of the upper bicuspids were devitalized by an operation made to get access to the antrum through the canine fossa. The pulps did not die immediately, but were so diagnosed a year after the operation was made. If such a result is possible, care should be exercised to not extend such opening sufficiently forward as to include these nerves.

New Method of Applying Aluminum Linings.—There have been several methods advocated for this purpose, but until now I have never found one which gave entirely satisfactory results. Having had almost invariable success with the following procedure, I give it to the readers ot the *Digest.* Pack and flask the case as usual, wet a piece of thin cotton cloth in hot water, and place it between the cast and rubber. Have the flask and rubber warm, put the case under the screw-press for a few minutes, take out and separate flask, remove cotton cloth, and apply the liquid aluminum mixture direct to the rubber. The chief advantages of this plan are, that the lining does not penetrate the plaster, and when vulcanized it comes out bright and clean, and very little burnishing finishes the case.—W. F. Magill, in *Dental Digest*.

LYSOL VERSUS SPIRIT OF SOAP FOR INSTRUMENT STERILIZATION.—Several years ago I made a research to determine the relative value of the various disinfectants for cleansing operating instruments, and came to the conclusion that a 5 percent solution of lysol was the most effective and practicable. As lysol has an odor which to some is unpleasant, and which is easily communicated to the atmosphere of the whole room unless particular precautions are taken, I was much pleased at the prospect of getting an antiseptic possessing all the advantages of lysol without its disagreeable odor. In order, however, to assure myself that spirit of soap is as reliable as lysol, I carried out a series of parallel tests between these two materials. Of these tests (fifteen in all), eleven resulted in favor of lysol, one in favor of spirit of soap, and three were inconclusive. From these results I was forced to the conclusion that spirit of soap is not equally reliable with lysol, so that, while I now use it for disinfecting the hands, I still adhere to the 5 percent solution of lysol for the sterilization of instruments.—W. D. Miller, in *Cosmos*.

